# Assessing adaptation and mitigation potential of roadside trees under the influence of vehicular emissions: A case study of *Grevillea robusta* and *Mangifera indica* planted in an urban city of India

**DOI:** 10.1371/journal.pone.0227380

**Published:** 2020-01-28

**Authors:** Hukum Singh, Mukta Yadav, Narendra Kumar, Amit Kumar, Manoj Kumar

**Affiliations:** Forest Research Institute, Dehradun, India; Universidade de Lisboa Instituto Superior Tecnico, PORTUGAL

## Abstract

The ever-increasing vehicle counts have resulted in a significant increase in air pollution impacting human and natural ecosystems including trees, and physical properties. Roadside plantations often act as a first defense line against the vehicular emissions to mitigate the impacts of pollutants. However, they are themselves vulnerable to these pollutants with varying levels of tolerance capacity. This demands a scientific investigation to assess the role of roadside plantation for better management and planning for urban sprawl where selected trees could be grown to mitigate the impacts of harmful pollutants. The present study assesses the impacts of vehicular emissions on the adaptation and mitigation potential of two important roadside tree species *i*.*e*. *Grevillea robusta* and *Mangifera indica* planted along roadsides in the capital city of Uttarakhand. Uttarakhand is one of the Indian Western Himalayan State and its capital city is situated on the foothills of Himalaya. The adaptation and mitigation potential were evaluated by studying the response of pollutants on the functional traits which drive the physiology of the trees. The CO_2_ assimilation rate, transpiration rate, stomatal conductance, water use efficiency (WUE), air pollution tolerance index (APTI), copper and proline accumulation, dust removal efficiency (DRE), leaf thickness and cooling created by plantation were studied to evaluate the response of trees exposed to roadside traffics. To compare the influence of pollutants, traits of trees grown in a control site with few or absence of vehicular movement were compared with the roadside trees. The control site represented part of a reserve forest where human interference is controlled and human-induced activities are prohibited. The vehicular frequency was found to modulate tree characteristics. The tree characteristics representing WUE, APTI, proline and copper accumulation, leaf thickness, cooling impact, and DRE were enhanced significantly, while the decreased CO_2_ assimilation rate was observed near roadside trees compared to the control site. We found both of the species to perform well to be used as one of the potential species for roadside and urban greening. However, there is a need to assess the potential of other species in reference to the present study.

## Introduction

The advent of technology has blessed us with a lot of inventions over the years and one of them was ‘the automobile'. However, the past 40 years have witnessed a tremendous increase in the number of vehicles as a result of the growing human population and developments have driven demands. This has resulted in the creation and expansion of roads adding to the soil, water, air, and noise pollution. Among all, particularly air pollution is of utmost concern along the roadside traffics. Recently, vehicular emission has been noticed to be one of the most concerns of urban air pollution. In such a scenario, it is the urban trees that could effectively mitigate the impacts of pollutants, especially air pollutants. Urban vegetation plays a significant role in combating air pollution. Trees act as an important and cost-effective solution to combat air pollution. However, different trees have varying levels of combating capacity distinguished with their adaptation and mitigation potential. This raises the question “Which are the most efficient trees appropriate to combat urban air pollution in an effective and efficient way?”. Urban green belts or urban roadside plantation act as a sink for particulate and gaseous emissions in a city and are often termed as “the lungs of the city”. Trees act as a sink for CO_2_ by fixing carbon during photosynthesis and storing carbon as biomass. Thus, the roadside plantations are expected to combat air pollution.

The roadside plantation is the first line of defense against urban vehicular pollution. The surface area of the leaves provides an opportunity for settling of suspended particulate matters while they also absorb gaseous pollutants [1]. However, in due course, they are themselves under various levels of stress manifested by the physiological response. On exposure to the airborne pollutants, most of the trees show immediate physiological response before the manifestation of the visible sign on leaves [2]. The tolerance capacity of the trees to these stresses defines their adaptation and mitigation potential. The mitigation potential of the trees is governed by several factors including anatomy, morphology, physiology, and biochemistry of plant tissues [3,16]. The vehicular movements affect the photosynthesis process, transpiration, stomatal conductance, proline content, and concentration of heavy metals in the plant tissue [3]. The air pollution adversely impacts the photosynthetic pigment and consequently reduces productivity. Leaf chlorophyll, carotene, proline, and proteins are found to be influenced adversely by air pollutants [4–8]. The air pollution influences stomatal functioning and leaf thickness of urban plantation [9,10]. Ascorbic acid is one of the strong indicators of stress and is found to be related to air pollution levels [11–13].

The potential to combat air pollution by various tree species still remains unknown for many species while its response under diverse conditions is not clearly understood [3]. Trees growing along the roadsides have been reported to exhibit many biochemical, morphological and anatomical changes as a result of higher concentrations of air pollutants. However, some trees have been reported to tolerate higher concentrations by modulating their physiological and biochemical traits. The more sensitive tree species act as biological indicators of air pollution. Some trees can resist fairly high levels of pollution and can be used effectively for combating air pollution [4]. Thus, it is important to screen trees that can act as indicators of stress under the influence of air pollution, and identify trees that can be used for mitigation of air pollution in an urban setup.

In this study, we characterized the physiological and biochemical response of trees to air pollutants which could be used to identify those having adaptation and mitigation potential to be adopted for urban greening. The two most widely grown species in the urban sprawls of Indian cities, i.e., *Grevillea robusta* and *Mangifera indica*, were selected under this study. We assessed several important parameters of these two species of trees to evaluate their adaptation and mitigation potential to combat air pollution [3]. This study would be helpful to assess the potential of other tree species following a similar approach.

## Materials and methods

### Location characteristics

**Fig 1:**
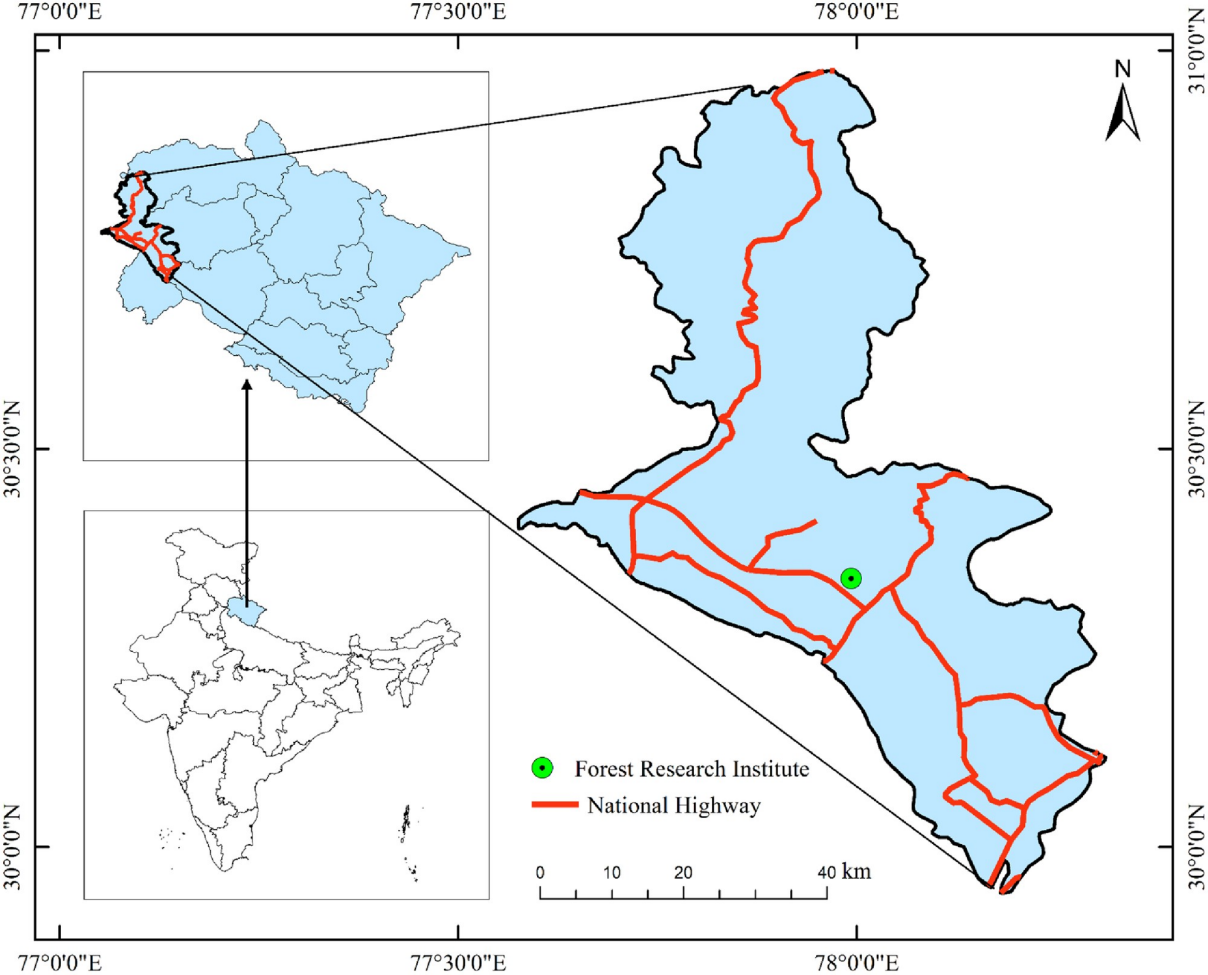
Experimental sites (national highway and Forest Research Institute) selected in the study.

The study was conducted in the Dehradun city of India (Fig 1). Dehradun is the capital city of Uttarakhand state, lying in the foothills of Himalayas. Dehradun is nestled in the mountain ranges of Himalayas. The area comes under the subtropical region and experiences the flavor of both temperate and tropical climates. The minimum air temperature ranges from 3.6 ° C to 29.4°C and maximum between 19.3°C to 35.3°C. The average air temperature of the last 20 years varied between 10.9° to 27.10° C, with the lowest temperature in January and highest during June. The relative humidity of the city ranged between 52.11 to 85.16% with minimum humidity in April and maximum in August. The 25-year rainfall data showed maximum rainfall during July (630.70 mm), while the minimum in December (2.80 mm). Sunshine hours per day averaged for the last 20 years was observed to be highest in the month of May (9.34 h day^-1^).

The economy and developmental activities of the city is growing very rapidly. The city has experienced a rapid increase in the number of vehicles which is added at a rate of 60,000 vehicles per year. In addition to this, being surrounded by the spiritual cities like Haridwar and Rishikesh; and hill stations like Mussoorie and Chakrata, the area has been continuously attracting national as well as international tourists escalating the vehicular traffic. Corollary to this, the air quality of the city has been deteriorated with an increase in the concentration of air pollutants, i.e., CO, Pb, NOx, SO_2_, CO_2_, VOCs, other toxic chemicals and particulate matter [3,14,15].

The assessment of impacts of air pollutants(considered in combined term to represent all of the pollutants such as CO, Pb, NOx, SO_2_, CO_2_, VOCs, other toxic chemicals and particulate matter) on trees was done by comparing the responses of trees under two different environments, viz., the control sites located within the reserve forest area of Forest Research Institute (FRI) exposed with least or no vehicular emissions and the urban roadside plantation exposed to heavy traffic emissions considered as the treatment sites. The roadside plantations of National Highway (NH-72, Haridwar-Dehradun-Ambala; 30°19’58.77’’- N 30°20’09.51”and 78° 00’19.31’’ E − 77°58^’^ 24.04’’E) passing through the Dehradun city were considered as treatment sites for this study. It was observed that, on average, almost 1000 vehicles pass per hour in both directions. The plantation of reserve forest area of FRI (a premier forestry research and education institute) (30° 19’59.7’’-30°20’44.6’’ N and 77°59’ 43.2’’ − 77°59’55.7’’E) had very less vehicular movement and we observed maximum number of vehicles passing at a rate of 40 vehicles per hour in both the directions. The strip randomized design was used for the selection of trees in the plantation area. The frequency of vehicular movement per hour was counted manually at both the sites for twenty-four hours.

### Monitoring adaptive physiological and mitigation response

The physiologically adaptation and mitigation plant functional traits such as leaf carbon assimilation rate (*A*- μmol CO_2_ m^-2^ s^-1^), water released through transpiration rate (*E-* mmol H_2_O m^-2^ s^-1^) and stomatal behavior in terms of stomatal conductance (*Gs-* mol H_2_O m^-2^ s^-1^) were measured using portable photosynthesis analyzer (LI-COR-6400 XT, Lincoln, NE USA). All these parameters were monitored from healthy leaf between 09:30 am to 01:30 pm in clear sky conditions. The water use efficiency (WUE- μmol CO_2_ mol^-1^ H_2_O) was accounted for as the ratio of carbon assimilation rate (*A*) to transpiration (*E*) [3,15–17]. The vapour pressure deficit of leaf was 4.7 and 6.04 at FRI and roadside conditions, respectively. Photosynthetically active radiation (PAR) was monitored and values of 1215 and 1277 μ mol m^-2^ s^-1^ at FRI and roadside, respectively, were found during the study period. All the parameters were monitored at the leaf level. Before measurement, the tree canopy of selected tree species in the roadside plantation was stratified into three strata i.e. bottom, middle and top. Each stratum was further divided into four directions. All the measurement was made from each stratum and direction totaling twelve sampling point in a canopy of a single tree. The aluminum bifurcated ladder with the flat top was used to reach the various stratum and direction of the canopy for measuring fluxes using a portable photosynthesis analyzer.

### Biophysical adaptive traits of leaf

The adaptive biophysical trait *i*.*e*. leaf thickness of selected species was monitored as one of the indicators of adaptation of vegetation towards abiotic stress. The leaves from each stratum were considered for monitoring leaf thickness. Leaf thickness was measured using a millimeter scale of digital vernier caliper. Two methods were adopted for measuring the leaf thickness, a) leaf thickness of individual leaf, b) leaf thickness of a leaf bundle consisting 10 leaves, the thickness of this bundle was then divided with the number of leaves (10 leaves) to obtain the leaf thickness of the single leaf. Both methods produced similar values of leaf thickness of a single leaf [3]. The values of leaf thickness presented in the results are the averaged values obtained using both the approaches.

### Monitoring dust removal efficiency—A mitigation trait of vegetation

The dust removal efficiency of leaves of the selected species as an indicator of mitigating urban air pollution was estimated. Three methods developed under this study were used. Mature and healthy leaves in each stratum were selected and marked (at petiole). The marked leaves were cleaned and left for 24 hours for the deposition of dust on leaves. After this period, these leaves were used for estimating dust removal efficiency by following three methods; a) the leaves were plucked and subsequently weighed at the experimental area using the digital balance. Then the leaves were cleaned using brush and tissue paper to remove the dust deposited over leaves. After that, the leaves were again weighed. The weight of leaves after dust removal was subtracted from the weight of leaves with dust deposition and expressed in gm dust leaf^-1^. The leaf area (cm^2^ leaf^-1^) of the same leaves was calculated using the graph paper method [17]. Then the dust removal efficiency of leaves was obtained using the ratio of dust load to leaf area which was expressed as gm cm^-2^ day^-1^ b) The plucked leaves were dipped in a beaker filled with 500 ml of distilled water with known weight at the experimental area itself for removal of dust particle intact on leaves. Then, the weight of the beaker containing dust particles in the water was measured using the digital balance in the experimental area itself. The weight of the beaker filled with 500 ml of distilled water was subtracted from the weight of the beaker containing dust particles to obtain dust deposition (gm) per leaf. The obtained dust deposition was divided with leaf area achieved through the graph paper method. The value of dust removal efficiency was mentioned as gm cm^-2^ day^-1^, and c) The plucked leaves were brought to the laboratory for further analysis. The leaves were directly weighed with dust intact using the digital balance. This weight was considered as the initial weight. Then the leaves were washed with distilled water to remove the dust accumulated over them and allowed to air dry for a few minutes for removing water droplets adhere on the leaf surface. Afterward, the leaves were again weighed and this weight was considered as the final weight. The difference of initial and final weight divided by the leaf area gave the amount of dust removal efficiency (gm cm^-2^ day^-1^) of the selected plantation species. The values of leaf area were used which obtained according to graph paper method. The values of dust removal efficiency presented in the results are the average of the values produced by the above methods.

### Development of air pollution tolerance index (APTI)

The study of a single parameter is not enough to provide a clear picture of the pollution-induced changes, Air Pollution Tolerance Index (APTI) which is based on various parameters, has been used to know tolerance levels of tree species [2]. The evaluation of the tolerance index of native trees of an area towards vehicular pollution will in turn help in the selection of trees for the green belt development in the affected areas so as to reduce the concentration of pollutants and hence their impacts on the environment and human health as well. Given that green corridors have to face stressed climatic conditions with intake of the high level of gaseous emission (SO_2_, CO, CO_2_, and NO_X_) and Suspended Particulate Matters (SPMs), it is important to select the species which are tolerant and at the same time capable ineffective adaptation and mitigation of pollution in limits of urban areas.

APTI determines the tolerance/sensitiveness of tree species towards air pollution. It is the most popular index used for screening tolerance tree species for this propose. Four physiological and biochemical parameters including leaf relative water content (RWC), ascorbic acid content (AAC), total leaf chlorophyll (TChl) and leaf extract pH were used to develop APTI. The APTI was computed from the leaves which were used for calculating dust removal efficiency. Under this study, for measuring leaf pH, 5 gm of fresh leaves were homogenized in deionized water. The slurry was subjected to centrifugation and the supernatant obtained was used for determining pH of the leaf using digital pH meter calibrated with the buffer solution of pH 4, 7 and 9.

Total chlorophyll content in leaf tissues was elucidated as per the method of Hiscox and Isralesham [18]. Fresh leaves (100 mg) were chopped into fine pieces and then transferred into vials filled with 7ml of Dimethyl sulphoxide (DMSO). The vials were incubated at 67°C for 30 minutes. The mixture was then filtered into a graduated test tube and the volume was made to 10ml using DMSO. The filtered sample was then used for monitoring Optical Density (O.D.) at 645 nm and 663 nm using Spectrophotometer. Total chlorophyll content of leaf tissues (mg g^-1^as fresh weight (F.W.)) was calculated using the following formula;

$$Total\;chlorophyll\;content(TChl)=\frac{\left[(20.2\times A645)+(8.02\times A663)\times V\right]}{a\times 1000\times w}$$

Where; A645 and A663 represent O.D. values measured at 645 nm and 663 nm, respectively. V = Final volume of extract, a = path length of the cells (1cm), and w = the weight of the sample taken.

For calculating relative water content (RWC), fresh weight of leaf was taken using digital balance, this was considered as the F.W. Then the leaf samples were dipped in water overnight and then again weighed. The weight so obtained was considered as the turgid weight (TW). Then the samples were oven-dried and then again weighed for measuring the dry weight (DW). The RWC of the samples was estimated using the following formula [30];

$$RWC(\%)=\frac{FW-DW}{TW-DW}\times 100\%$$

The method of Mc Cormick and Greene [19] was adopted for elucidating ascorbic acid content in leaf tissues. Fresh leaf tissues (0.2 gm) were homogenized in 2 ml of Trichloroacetic acid (TCA) using pestle mortar. The mixture was centrifuged at 2000 rpm for 10 minutes. A pinch of activated charcoal was added and then again centrifuged for 10 minutes. The supernatant (0.5 ml) was taken in a test tube and then 1.5 ml TCA, 0.5ml of 2,4-Dinitrophenylhydrazine (DNPH) and 2 ml of thiourea was added to it. After that, the mixture was kept in a water bath at 37°C for 3 hours. Then 2.5 ml H_2_SO_4_ was added to it keeping in an ice-tray. After incubation of 30 minutes at room temperature, absorbance was read at 540 nm against the blank. The ascorbic acid content (mg g^-1^ F.W.) was determined with the help of a standard curve prepared using the absorbance of the working stock solution of known concentrations.

Finally, APTI was derived using equation formulated by Singh and Rao [20];

$$APTI=\frac{\left[A(TChl+P)+R\right]}{10}$$

Where, APTI = Air Pollution Tolerance Index, A = Ascorbic acid (mgg^-1^ F.W.), TChl = Total chlorophyll content (mg g^-1^ F.W.), P = pH and R = Relative water content (%).

### Estimation of proline and copper accumulation in leaf tissues

Proline is one of the key stress as well as an adaptive indicator of the tree system. A sample of fresh leaves (0.5 gm) was homogenized in 10 ml of 3% aqueous Sulphosalicylic acid. The homogenate was filtered through Whatman filter paper (no. 2). The filtered sample (2 ml) was taken in a test tube and 2 ml of glacial acetic acid and 2 ml of ninhydrin solution was added to it. The mixture was boiled in a boiling water bath for 1 hour. The reaction was terminated by keeping the tube in ice-bath. Then 4 ml of toluene was added to the reaction mixture and stirred well. The upper red-colored toluene layer was separated using a micropipette and kept at room temperature. The absorbance of red color was measured at 520 nm using a spectrophotometer. A series of the standard with pure proline was run in a similar way and a standard curve was prepared. The amount of proline in a sample was estimated using the standard curve [21].

To understand the accumulator nature of tree species towards heavy metal (only Copper, “Cu” considered for this study), the copper content in leaf tissues was investigated. Samples of fresh leaves were washed thoroughly with 0.1M HCl and then with demineralized water and subsequently oven-dried at 60°C. Oven-dried leaf sample (1 gm) was taken in a 100 ml conical flask and then a 15 ml Di-acid mixture of HNO_3_ and HClO_4_ (10:4) was added into each flask and further subjected to digest using a hot plate. The digested sample was turned in to white powder form. The digested white powder samples were transferred into 50 ml volumetric flasks and further added 40 ml distilled water. This solution was then filtered through filter paper (Whatman–no 42) and final volume made-up to 50 ml with distilled water. The samples were kept in the refrigerator at 4°C until the analysis of metal concentration. After that, the copper content of the samples was determined using the flame atomic absorption spectroscopy (FAAS). The limit of detection (LOD) for Cu was 0.001 mg kg^-1^. FRI laboratory follows standard guidelines of quality control and assurance for the chemical analysis including heavy metals analysis, nutrient analysis and other biochemical analysis of various samples such as the leaf, water, air, and dust particles. During Cu estimation in leaf tissues, standardized quality assurance procedure and precaution were followed for ensuring the reliability of results. Analytical quality was assured by repeated measurement of the blank procedure, reagent blank, duplicate samples and certified reference material of copper (Sigma-Aldrich, Inc.; TraceCERT^®^; Cat No. 38996; 1000 mg L^-1^ Cu in 2% nitric acid). All the reagents of analytical grade were used throughout the analysis. All glassware was washed with dilute HNO_3_ acid and deionized water before use to prevent contamination.

### Analysis of local cooling created by plantation species

The difference between air temperature of the outside of the canopy and below the canopy of tree species was considered as local cooling created by the selected plantation at both the experimental area. For monitoring the local cooling created by the tree species, it is important to decide the exact point where the air temperature is to be measured. During the day, when insolation impinges on tree canopy, a part of solar radiation is absorbed by the canopy increasing the air and leaf temperatures within the canopy and the other part reaches the ground-penetrating through the canopy, which increases the temperature of soil surface. As the soil surface warms, it not only conducts heat deeper into the soil but also radiates back into the air augmenting the air temperature above the ground surface mostly up to the 1.5 m. The air temperature up to 1.5 m above the ground is affected utmost by the radiation from soil [22,23]. We monitored the temperature at 1.5 m above the ground surface to minimize this impact. The temperature was monitored within the canopy as well as outside the canopy to compare the influence of canopy on the ambient temperature. The temperature was monitored between 12:00 noon to 2:00 pm on a fair-weather four times in a month. All the directions of the sample tree were covered to monitor air temperature. Then, an average of all the monitored values of temperature was taken to calculated cooling impact based on the temperature between outside the canopy and under the canopy of both the species at both the experimental sites [24].

#### Statistical analysis

The data were statistically analyzed using GENSTAT to know the significant difference between observed traits of both the experimental areas. The data are presented as the mean of the observation along with standard error.

## Results

### Physiological adaptation and mitigation response of urban roadside plantation towards air pollution

**Fig 2:**
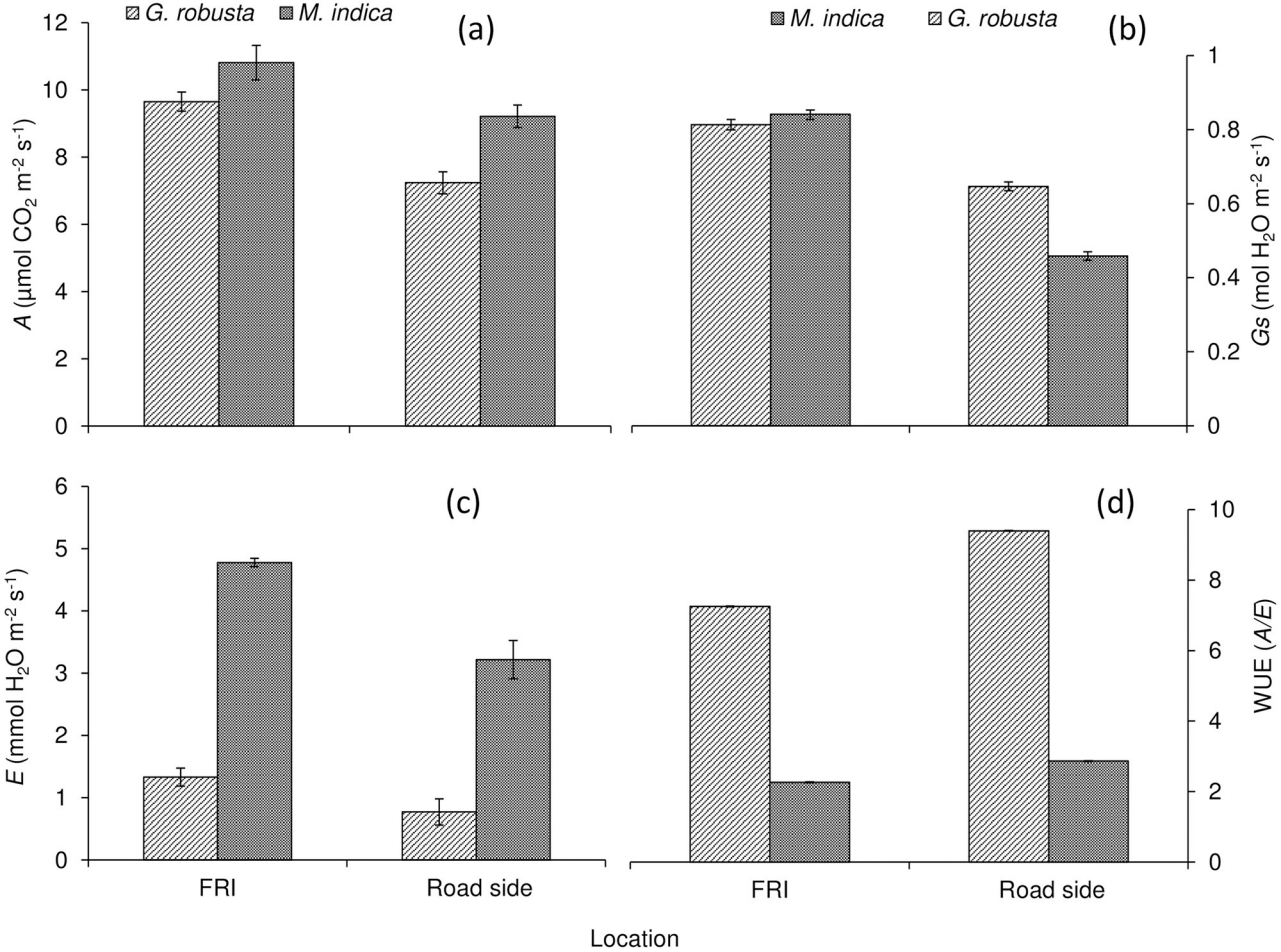
Impact of air pollution on adaptation and mitigation behaviour of urban roadside plantation.

The response of urban plant functional traits (UPFTs) regulating adaptation and mitigation potential of the urban roadside plantation is presented in Fig 2. The frequency of vehicular movements was found to be significantly altering the adaptation and mitigation potential of the selected plantation (*Grevillea robusta* and *Mangifera indica*) along the roadside of the urban area. The air pollution mitigation parameters *i*.*e*. CO_2_ assimilation rate (*A*) of *Grevillea robusta* and *Mangifera indica* declined significantly (*p* = ˂0.05) for roadside plantation compared to FRI where the vehicular movement was least (Figs 2a and 6). It was interesting to report that *A* declined (~15%) at the roadside plantation of *M*. *indica* (9.21±0.39 μmol CO_2_ m^-2^ s^-1^) compared to FRI (10.81±0.52 μmol CO_2_ m^-2^ s^-1^). However, highest reduction (~26%) of *A* was investigated for the plantation of *G*. *robusta* at the roadside (7.23±0.29 μmol CO_2_ m^-2^ s^-1^) rather than FRI (9.66 ±0.44 μmol CO_2_ m^-2^ s^-1^).

There was an utmost drop in G*s* reported for *M*. *indica* at the roadside plantation of both the species (Figs 2b and 6). The roadside plantation of *M*. *indica* expressed lower G*s* (0.38±0.01 mol H_2_O m^-2^ s^-1^) compared to FRI (0.84±0.02 mol H_2_O m^-2^ s^-1^) which showed a reduction ~43%. Conversely, G*s* of *G*. *robusta* revealed less reduction (~21%) than *M*. *indica* at the roadside, which represented diminution at the roadside (0.64±0.01 mol H_2_O m^-2^ s^-1^) when comparison made with FRI (0.81±0.01 mol H_2_O m^-2^ s^-1^).

The physiological adaptive traits of plantation especially stomatal behavior in terms of stomatal conductance (*Gs*) and water use efficiency (WUE) was noted to be distorted because of vehicular movements (Fig 1). It was reported that more vehicular frequency at roadside induced to decline *E* (~33%) of *M*. *indica* (3.21±0.01 mmol H_2_O m^-2^ s^-1^) compared to plantations exposed to less vehicular movements *i*.*e*. FRI (4.78±0.18 mmol H_2_O m^-2^ s^-1^). Although, *G*. *robusta* exhibited the highest reduction in *E* (~43%) (0.77±0.01 mmol H_2_O m^-2^ s^-1^) than *M*. *indica* at the roadside when compared to the counterpart plantation, FRI (1.33±0.02 mmol H_2_O m^-2^ s^-1^) (Figs 2c and 6).

It was surprising that WUE, an important adaptive trait of trees was significantly (*p* = <0.05) improved at the roadside plantation of both the selected tree species (Figs 2d and 6). Maximum improvement in WUE was reported for *G*. *robusta* (~30%) compared to *M*. *indica* (~27%) at the roadside plantation. Among both the species, *G*. *robusta* was found to be more adaptive in terms of WUE at urban roadside environment having a value of 9.40±0.22 μmol CO_2_ mol^-1^ H_2_O compared to the sites of FRI which had the value of 7.25±0.14 μmol CO_2_ mol^-1^ H_2_O. On the other hand, *M*. *indica* illustrated a similar pattern as shown by *G*. *robusta* however the magnitude differed significantly. *M*. *indica* received highest WUE (2.90±0.02 μmol CO_2_ mol^-1^ H_2_O) at the roadside and least at FRI (2.26±0.02 μmol CO_2_ mol^-1^ H_2_O).

### The response of adaptation at the biophysical level of the leaf towards air pollution

Leaf thickness, one of the important biophysical parameters of leaves is very sensitive which is modulated for adapting to air pollution as well as other abiotic stresses and changing climatic conditions. Under the present study, leaf thickness was found to be increased significantly for trees growing along the national highway/road in the vicinity of the urban area in contrast to control sites of FRI (Figs 5a and 6). The plantation of *G*. *robusta* significantly boosted the average leaf thickness of 0.45±0.02 mm leaf^-1^ (~55%) compared to the plantation of FRI (0.29±0.01 mm leaf^-1^). Although, leaf thickness of *M*. *indica* had achieved an increment of (~31%) 0.55±0.03 mm leaf^-1^ when compared with the counterpart plantation of FRI (0.42±0.02 mm leaf^-1^). The findings of leaf thickness showed that the frequency of vehicular movements led to increasing leaf thickness of the selected plantation, which could be one of the important adaptive responses of plantations to air pollution caused by the increase of vehicular frequency on the national highway in the urban area.

### Leaf response towards dust removal efficiency (mitigation nature) of plantation under air pollution circumstances

The dust removal efficiency (DRE) of the plantation was significantly improved at roadside rather than the least vehicular movements at FRI (Figs 4b and 6). It was surprising to observe that plantation of *G*. *robusta* exhibited better DRE (~224%) of about 3.67±0.08 mg cm^-2^ day^-1^ for the roadside plantation in contrast to FRI (1.13±0.04 mg cm^2^ day^-1^). In the present study, *M*. *indica* was found to be attained more (~158%) DRE approx 2.67±0.03 mg cm^-2^ day^-1^ at the roadside while lower for the plantation of FRI (1.0±0.01 mg cm^-2^ day^-1^) where vehicle traffic is less.

### The response of air pollution tolerance index (APTI) and other associated parameters of the plantation to urban roadside air pollution

**Fig 3:**
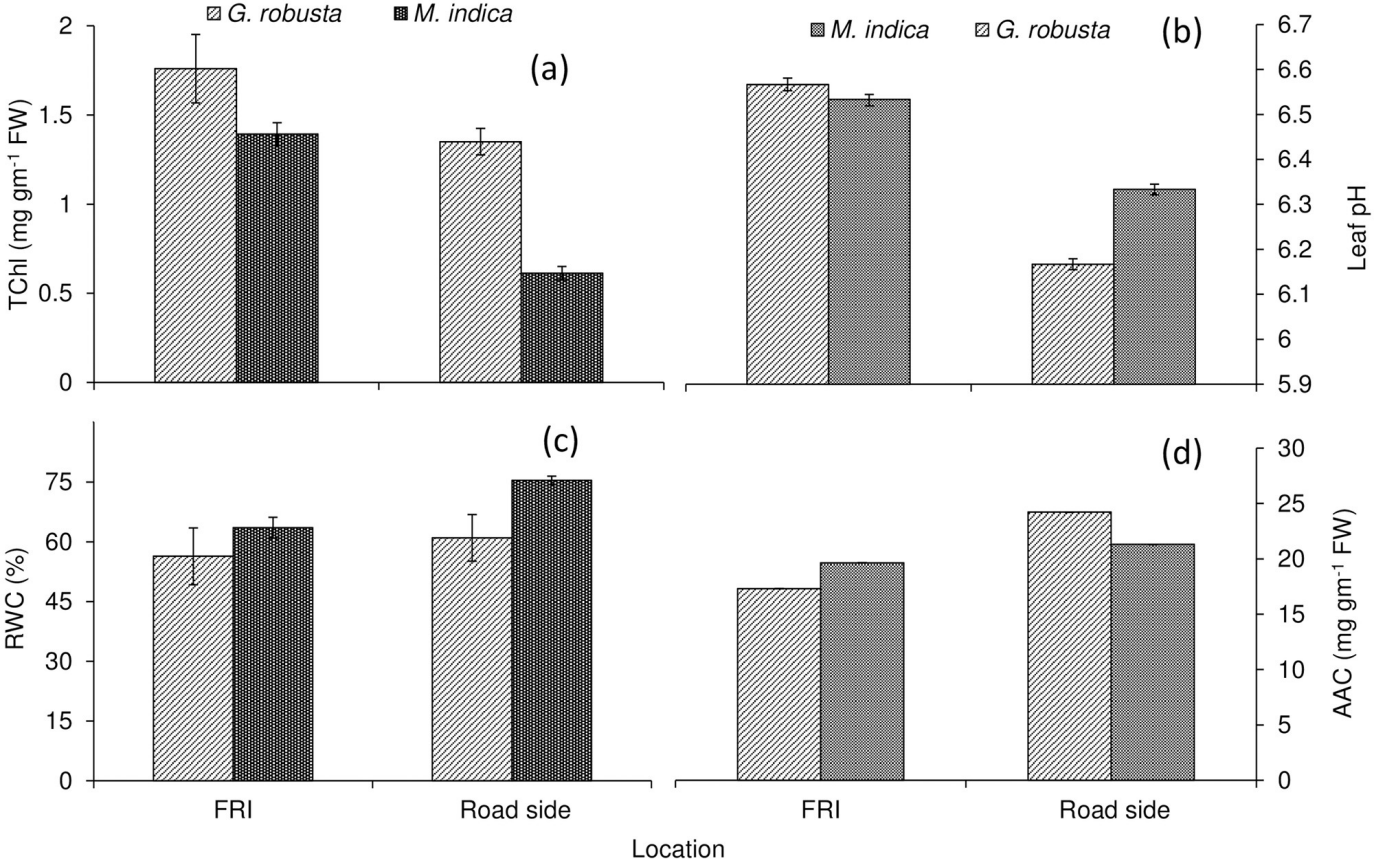
Impact of air pollution on RWC and biochemical response of urban roadside plantation.

**Fig 4:**
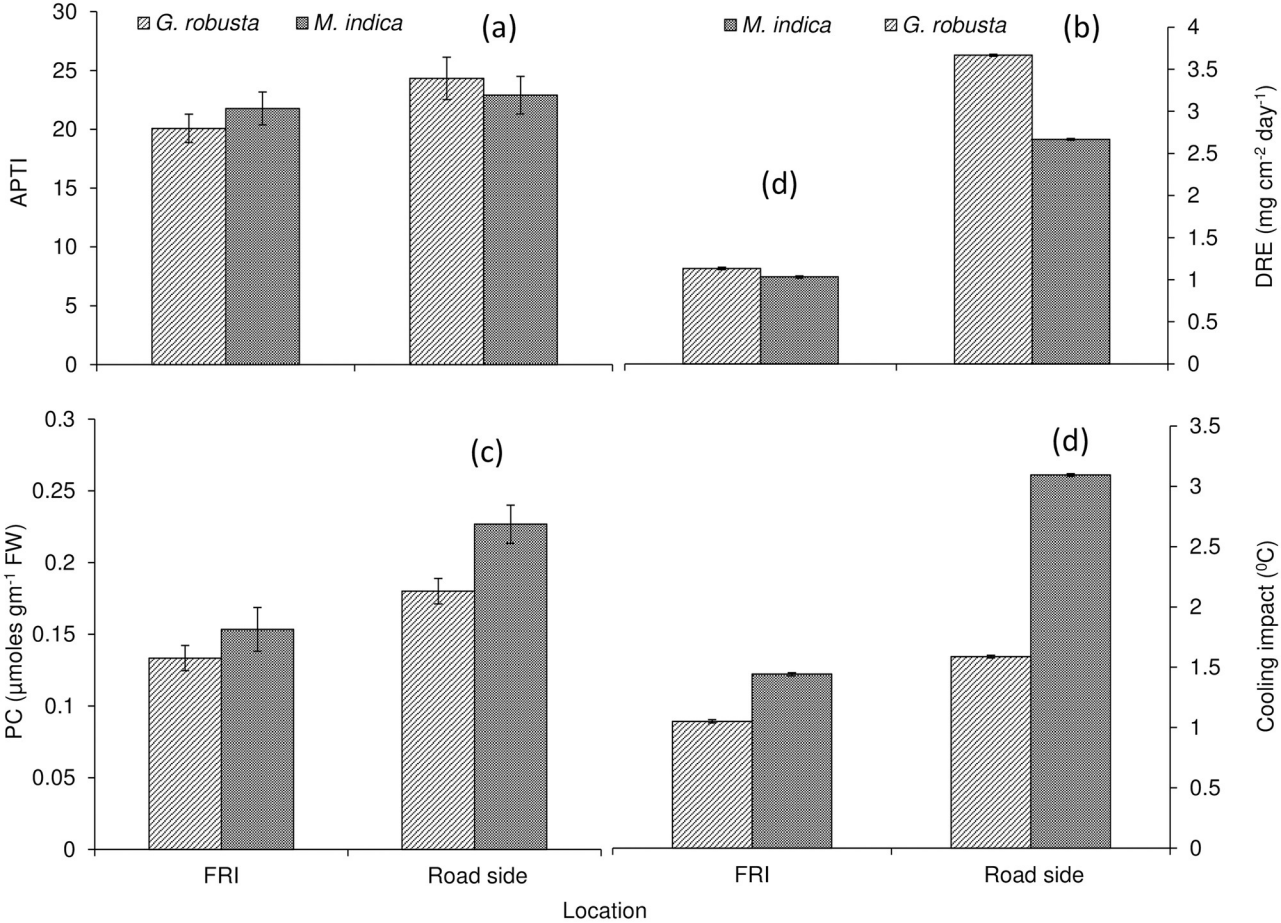
Impact of air pollution on adaptation and mitigation nature of urban roadside plantation.

APTI is a key plant parameter that indicates the tolerance or sensitivity of tree species toward air pollution caused by vehicular movements and other interventions. In the present study, average APTI was maximum found for *G*. *robusta* followed by *M*. *indica* (Figs 4a and 6). The leaf pH of *G*. *robusta* showed more variation between plantation of roadside (6.16±0.52) and FRI (6.57±0.61) whereas pH of *M*. *indica* varied from 6.33±0.54 (roadside) to 6.53±0.59 (FRI) (Figs 3b and 6). Urban roadside air pollution was profoundly decreased total chlorophyll content (TChl) in the plantation of both the species (Figs 3a and 6). The highest reduction (~59%) in TChl of *M*. *indica* plantation of roadside (0.61±0.01 mg g^-1^ F.W.) was reported in comparison to the FRI plantation (1.40±0.02 mg g^-1^ F.W.). The TChl of *G*. *robusta* varied from 1.35±0.02 mg g^-1^ F.W. at the roadside plantation to 1.76 ±0.03 mg g^-1^ F.W. of FRI with a reduction of ~24% at the roadside. The RWC in leaf tissues of both the species was increased at roadside plantation compared to the least air pollution within the premises of FRI (Figs 3c and 6). RWC for *G*. *robusta* trees was computed more (~61%) at the roadside plantation compared to FRI (~56%). A similar trend was shown by *M*. *indica* with higher RWC at the roadside (75.34%) rather than FRI (63.53%). *M*. *indica* exhibited higher RWC at both the experimental area compared to *G*. *robusta*. Both the species grown along the roadside was experienced more accumulation of Ascorbic acid content (AAC) in response to heavy vehicular pollution as compared to the FRI (Figs 3d and 6). The AAC for *G*. *robusta* was recorded 17.31±1.27 mg g^-1^ FW for the plantation in the FRI whereas 24.22±1.98 mg g^-1^ FW for those plantations grew on roadside conditions which showed an increment ~39.9%. However, the increase in AAC for *M*. *indica* was less with the value of 19.64±1.15 mg g^-1^ FW in FRI trees while 21.31±1.17 mg g^-1^ FW in roadside trees thus showed an increase of only 8.5%.

### Proline and copper accumulation in leaf tissues in response to urban air pollution

Proline accumulation in leaf tissues of both the species was significantly (*p* = ˂0.05) enhanced for the roadside plantation when compared to FRI (Figs 4c and 6). The highest proline accumulation (~48%) was elucidated for the plantation of *M*. *indica* at the roadside (0.23±0.02 μ mol g^-1^ FW) and lowest at FRI (0.15±0.01). *G*. *robusta* accumulated less proline in leaf tissues when compared to *M*. *indica*. Air pollution was reported to enhance leaf proline at the roadside plantation of *G*. *robusta* (0.18±0.02) with least at FRI (0.13±0.01) with an increase of ~35% at the roadside plantation.

**Fig 5:**
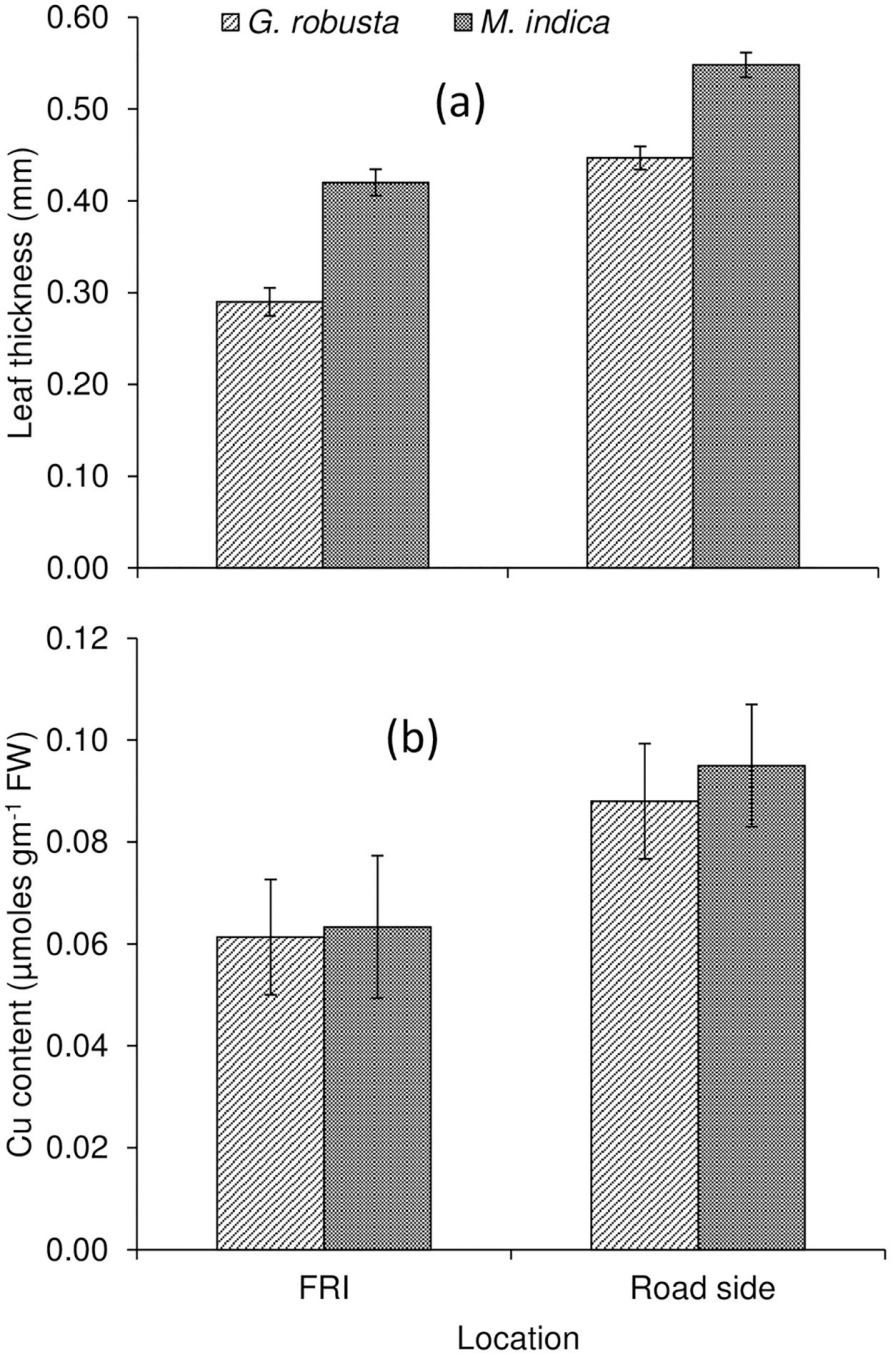
Impact of air pollution on leaf thickness and copper content in leaf tissues of urban roadside plantation.

**Fig 6:**
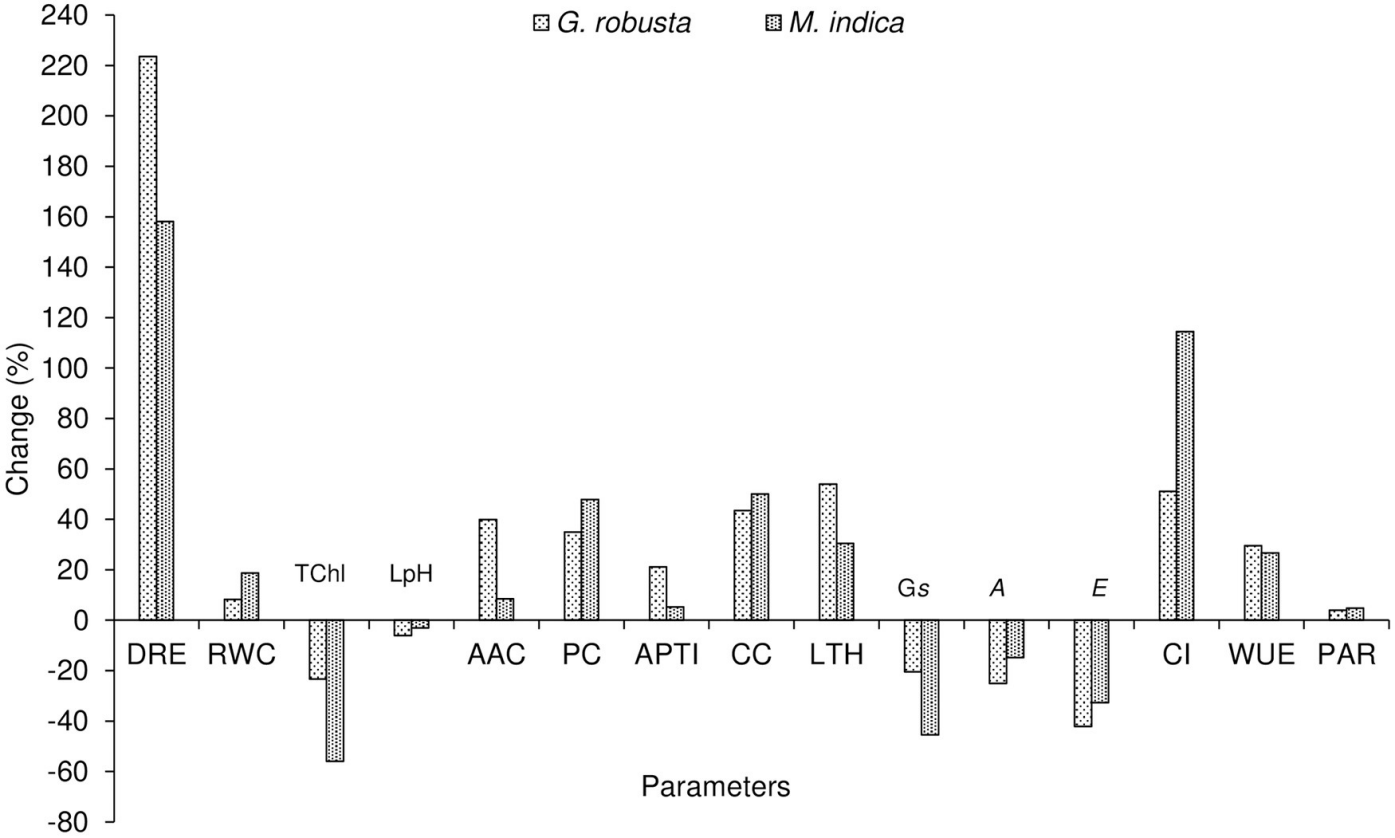
Impact of air pollution on magnitude of adaptation, mitigation and other associated traits of urban roadside plantation. (DRE: Dust removal efficiency; RWC: Relative water content; TChl: Total chlorophyll content; LpH: Leaf pH; AAC: Ascorbic acid content; PC: Proline content; APTI: Air pollution tolerance index: CC: Copper content; LTH: leaf thickness; G*s*: Stomatal conductance; A: carbon assimilation rate; E: Transpiration rate; CI: Cooling impact; WUE: Water use efficiency, and PAR: Photosynthetically active radiation).

Copper content (Cu) was significantly improved in leaf tissues of the plantation at roadside compared to FRI (Figs 5b and 6). There was an increment (~50%) in Cu content (0.095±0.002 μmol gm^-1^ FW) in the leaf of *M*. *indica* compared to their counterparts grown in FRI (0.063±0.002 μmol gm^-1^ FW). *G*. *robusta* exhibited a similar trend with more increment (45%) at the roadside (0.09±0.02 μmol gm^-1^ FW) than FRI plantation (0.061±0.001 μmol gm^-1^ FW).

### The cooling impact created by plantation species

It was important to note that vehicular movement found to have a significant impact on cooling provided by both the species at roadside plantation (Figs 4d and 6). The higher cooling was provided by *M*. *indica* than *G*. *robusta* at the roadside. *M*. *indica* created more cooling at the roadside compared to the FRI by ~120%. For *G*. *robusta*, maximum cooling was recorded at the roadside which was greater than the FRI by ~51%.

## Discussion

### Physiological adaptation and mitigation response of urban roadside plantation to air pollution

The vehicular movements altered the adaptation and mitigation potential of the urban plantation. The physiological adaptation traits manifested by CO_2_ assimilation, water loss through transpiration rate and stomatal opening and closing in the form of stomatal conductance was reduced for the trees near roadside species. This was due to the deposition of particulate matters on the leaves. Particulate matters partially or fully close the stomatal pore that regulates transpiration and the uptake of carbon dioxide for photosynthesis [3,16]. Conceptually, stomatal conductance depends on various environmental factors, position, and age of the tree leaves. Any interruption in stomatal behavior changes the gaseous exchange by reduced CO_2_ assimilation rate (photosynthesis) and the exchange of water (transpiration). On the other hand, it was observed that the leaf surface of trees along with the roadside deposited more dust particles. The deposition of dust interferes with the gas exchange between the leaf and ambient air. The deposition also causes a reduction of leaf stomatal conductance which directly influences transpiration, ultimately modulates the adaptation mechanism for producing tree biomass [25]. In addition to these, water use efficiency of trees was enhanced at roadside trees compared to control sites of FRI reserve forest plantations. The reduced transpiration rate due to suppressed stomatal conductance leads to an increase in physiological water use efficiency.

The results reveal an intriguing outline of the fate of emissions from vehicular traffic. It was observed that leaves regulate CO_2_ assimilation at different rates under different deposition of particulate matters. The transpiration rate is also regulated under different deposition leading to an alteration in water use efficiency which was quite similar to the findings of Singh et al [3]. The physiological traits studied by us could be used to define the adaptation and mitigation potentials of trees. For example, the foliar surface of urban roadside trees acts as a sink for dust, particulate matter deposition, and other pollutants. The deposition on leaves alters morpho-physiological and biochemical responses of trees with structural and functional changes. This eventually affects the CO_2_ assimilation rate and water use efficiency [26]. Air pollutants at the roadside environment directly affect photosynthesis, respiration, transpiration, water use efficiency and stomatal conductance. All the modulations in the various physiological and biochemical processes are happened for acclimatizing or adapting the altered environment so that urban roadside pollution could be mitigated or cleaned by a selective plantation approach. The results of the present study were parallel to the study conducted by various researchers on the roadside plantation [3,27].

### The response of adaptation at the biophysical level of the leaf towards air pollution

The leaf thickness of both the species has been found to be more in the trees growing along the roadside than the trees growing in the FRI campus. More leaf thickness has been observed in *M*. *indica* as compared to *G*.*robusta*. However, the percentage increase in leaf thickness at the polluted site as compared to the control site was found to be more in *G*. *robusta* than that in *M*.*indica*. Increased leaf thickness of roadside trees would be due to the waxy cuticle and the leaves are sticky in nature in which dust particles have adhered. Thicker the cuticle layers of the leaf surface, the slower the transpiration rate which directly affects the stomatal behavior of the leaf. It was also reported that the increase of the leaf thickness in polluted trees indicates a high resistance of these species to heavy vehicular pollution [10] and can be seen as an adaptation mechanism to stress. The leaf thickness under the present study was supported by various workers [28,29].

### Leaf response towards dust removal efficiency (mitigation nature) of plantation under air pollution circumstances

Leaf’s response towards dust deposition was found to increase in both the species of roadside conditions having more vehicular movement as compared to fewer movements at the FRI. The settlement of dust particles on the leaf surface mainly depends on the phyllotaxy, smoothness, shape, petiole length as well as the position of leaf in the tree [30]. *G*. *robusta* has accumulated significantly more dust than *M*. *indica* at both the sites. The increased dust deposition on the leaf surface could be the result of smoother surface and geotropic position of the leaves of *M*. *indica* as compared to the rough surface and geo-parallel position of the leaves of *G*. *robusta*. Also, *G*. *robusta* leaves have a larger surface area than that of *M*. *indica* and leave with more surface area are more efficient to trap the dust particles [1].

### The response of air pollution tolerance index (APTI) and other associated parameters of the plantation to urban roadside air pollution

Under the current study, the average APTI maximum was found for *G*. *robusta* followed by *M*. *indica*. APTI was estimated to understand the response of tree species towards air pollution at physiological and biochemical levels. With a very little difference in the APTI values both the species can be considered intermediately tolerant to the vehicular pollution, according to the categorization (APTI value <1 = very sensitive, 1–16 = sensitive, 17–29 = intermediate and 30–100 = tolerant) [10]. It was reported that species having higher APTI values are considered more tolerant of air pollution than the ones having lower value [2]. The trees with high and low APTI can serve as tolerant and sensitive species respectively [31]. A similar study was also supported by Sharma et al [16] who stated that vehicular emission has become the main cause of air pollution. The green belts can be proper ecological mean to control air pollution. The highest APTI was recorded for *Toona cilliata* followed by *Leucaena leucocephala*, *Dalbergia sisso*, *Grewia optiva*,and *Ficus palmata*. The tolerance level for shrubs was recorded highest for *Adhatoda vasica* followed by *Murraya koenigii*, *Carissa opaca* and *Debregeasia hypoleuca*. They further suggested that among tree species *Toona cilliata* and *Leucaena leucocephala* and in shrub species *Adhatoda vasica* and *Murraya koenigii* are suitable for plantation along the National Highway.

The APTI depends on several other parameters such as total chlorophyll, pH of leaves, relative water content and ascorbic acid. Total chlorophyll content (TChl) of both the species showed significantly reduced in the roadside conditions as compared to the FRI campus side. It might be due to the degradation of chlorophyll into pheophytin by the loss of magnesium ions. Leaf surface of trees in heavy polluted areas degraded chlorophyll content thereby reducing photosynthesis [5]. Thus any modification in chlorophyll concentration might change the morpho-physiological and biochemical activities of a particular species growing in roadside conditions. A similar study has been reported by a number of workers [32,33]. It is also reported that a reduction in the chlorophyll content due to the dissolution of the dust particles in cell sap resulted in the degradation of the leaf pigment [7,34].

RWC was recorded more in roadside trees as compared to the FRI. High water content in trees ensures the maintenance of the physiological balance under stresses such as air pollution. Paul et al. [35] reported that greater is the exposure of trees to pollution stress, larger is the quantity of relative water content. The trees with high relative water content under polluted conditions were more tolerant of air pollution [36–38]. Mohammad et al. [39] also observed that relative water content was high in tree species growing at polluted sites and it further increased the drought and stress tolerance in trees. Lohe et al. [40] reported that the tolerance of terrestrial trees to air pollution was studied and it was recorded that the trees with high relative water content under polluted conditions were considered more tolerant of pollution.

AAC was significantly enhanced at roadside compared to the FRI plantation. Higher AAC in the FRI might be linked to higher pH in FRI plants. Higher pH favors converting hexose sugar to ascorbic acid [2]. Ascorbic acid plays a key role in carbon fixation, synthesis of the cell walls and cell division. Apart from this, it is also considered as a natural antioxidant and prevents the destructive effect of pollutants in tree tissue [41]. It was also reported that high AAC favors pollution tolerance in trees and is involved in the defense mechanism of trees [11]. Jyothi and Jaya [12] reported that tree species growing along roadside showed higher AA content and tolerance to the heavy pollutant.

Leaf pH acts as a biochemical indicator for sensitivity to air pollution, lower pH indicated higher pollution [42]. Leaf pH of roadside trees of both *G*. *robusta* and *M*. *indica* exhibited a slightly acidic pH as compared to the leaf pH of FRI campus trees. The reason might be the presence of NO_2_ and SO_2_ in the ambient air causing a change in pH of the leaf sap towards the acidic side [41]. In low pH, photosynthesis is also diminished in trees and interestingly trees are more prone to air contamination at low pH, whereas the trees having pH near 7 are more tolerant of the air contamination. In the area of acidic pollutants, there is a sudden drop in leaf pH. As a result, the increase in leaf pH provides greater resistance to species against air contamination. Acidic pH movement in the cell sap of leaf alters the productivity of the transformation of hexose sugar to ascorbic acid in the area of acidic pollutants [43].

### Proline and copper accumulation in response to urban air pollution

Proline has been considered as one of the biochemical indicators of stress in tree systems. The present study revealed that proline accumulation significantly enhanced in the leaves of the trees growing on the roadside as compared to the FRI. During various stresses, proline accumulation in leaf tissue protects the tree species. Therefore, it acts as an indicator of the adaptation potential of the trees besides that heavy pollution impact on various species on the roadside is very high [44,45]. Vehicular pollutants were responsible for the deleterious effect on the tree species by the production of reactive oxygen species which consequences in peroxidative destruction of cellular constituents. It is well-established facts that proline act as a free radical scavenger to protect trees away from damage by oxidative stress. Accumulation of proline in trees is a physiological response to osmotic stress [8]. Studies have shown an increase in proline content of leaves under stress conditions [3]. It was also stated that proline accumulation in leaf tissues under the stressed environment is an excellent indicator for screening tolerant species towards pollution stress [46]. All these studies support the finding of the present study.

The roadside plantations also play a critical role in the uptake of heavy metals from the atmosphere where accumulation has come from the combustion of vehicular fuel during movements. The tree species that act as hyper-accumulators are being preferred for plantation purposes along the national highway/roadside for increasing green cover for mitigating air pollution. Under the present study, therefore tree species along the roadside of the urban area are considered an alternative to removing air pollutants from the air including heavy metals and thus improving the air quality. Roadside trees accumulate various levels of heavy metals in different parts of trees such as leaves, roots, trunks, barks or fruits. These accumulations are appropriate to use in order to monitor the heavy vehicular air pollution [47–49]. Due to anatomical and physiological differences, the uptake, accumulation, and translocation of the copper metal varies from species to species. The increase in the copper (Cu) content in the leaves of the roadside trees can be attributed to the fact that the roadside trees face more vehicular movement and are therefore more exposed to exhaust fumes of motor vehicles, abrasion of tires and road surfaces, wear of brake linings, wear of engine parts and corrosion of various vehicle components which contains copper [11]. It has been reported that Cu deficiency and excess influence many enzymes that catalyze oxidation and reduction reactions [50,51]. Depending upon the tree species the suitable Cu concentrations for trees range from 2 to 20 ppm and the phytotoxic level is nearly 30 ppm [55]. The current finding of Cu in leaf tissues of both the species was in the line of the prior discussed works of various researchers.

### The cooling impact created by plantation species

Trees are known to produce a cooling effect because of the canopy shade and the process of transpiration. When the ambient temperature rises, trees lose water vapours, just like sweating in humans, and thus cool the ambient atmosphere [52]. The present study showed an increment in cooling at roadside plantation compared to FRI. It has been reported that vegetation reduces air temperature due to shade under the canopy i.e. blocking the direct heat radiations, as well as by moderating solar heat gain through evapotranspiration of the trees [24]. The cooling results of this study are corresponding to the findings of Abdel-Aziz [53] who reported that temperature reduction under tree shades varied from 1°C to 5°C while Lin and Lin [23] recorded temperature reduction from 0.64°C to 2.52°C below the canopy of the trees. Literally, tree canopy establishes liking among the terrestrial environment and the atmosphere, which is responsible for regulating various bio-physiological courses like interception of solar radiation, precipitation, photosynthesis, transpiration, respiration, etc [54]. The canopy hinders the flow of solar energy and exhibits physiological performance, which produces cooling in the atmosphere [24].

## Conclusion

The urban green space when planted with selected trees having adequate potential to combat air pollution could be one of the effective ways to reduce ambient air pollution of the city. The roadside plantations act as the first defense line against vehicular pollution of the city. The vehicular emissions modulate the physiological traits of the trees while different species may have different responses. Selecting a tree species that are more tolerant and efficient to combat air pollution could be useful for plantation along the roadside however there is little information available to select the appropriate trees. The trees may have different responses to the pollutants located in different geographical areas. Thus it becomes important to investigate responses in varying diverse physiographic locations to select trees that could be used to combat air pollution in a given geographical area. We investigated the response of two important species, viz., *Grevillea robusta* and *Mangifera indica* against air pollutants for an urban city. The comparison was made between the responses of the roadside trees, exposed to heavy traffic with a higher concentration of air pollutants, with the responses of trees planted in a reserve forest, where there was very little vehicular movement thus low level of air pollutants. The investigation traces the tolerance capacity of the selected trees against air pollutants and could be used to select other tree species that have the potential to combat air pollution.

## Supporting information

S1 Data (XLSX)
